# Self-transcendent experiences as promoters of ecological wellbeing? Exploration of the evidence and hypotheses to be tested

**DOI:** 10.3389/fpsyg.2022.1051478

**Published:** 2022-11-14

**Authors:** Amy Isham, Patrick Elf, Tim Jackson

**Affiliations:** ^1^Centre for the Understanding of Sustainable Prosperity (CUSP), Centre for Environment and Sustainability, University of Surrey, Guildford, United Kingdom; ^2^School of Psychology, Swansea University, Swansea, United Kingdom; ^3^Middlesex University Business School, Centre for Enterprise and Economic Development Research (CEEDR), Middlesex University, London, United Kingdom

**Keywords:** ecological wellbeing, self-transcendent experiences, psychedelics, awe, mindfulness, flow

## Abstract

In recent years, much has been written on the role of different mental states and their potential to influence our way of thinking and, perhaps more importantly, the way we act. With the recent acceleration of environmental and mental health issues, alongside the limited effectiveness of existing interventions, an exploration of new approaches to deliver transformative change is required. We therefore explore the emerging potential of a type of mental state known as self-transcendent experiences (STEs) as a driver of ecological wellbeing. We focus on four types of STEs: those facilitated by experiences of flow, awe, and mindfulness, as well as by psychedelic-induced experiences. Some of these experiences can occur naturally, through sometimes unexpected encounters with nature or during immersion in every-day activities that one intrinsically enjoys, as well as through more intentional practices such as meditation or the administration of psychedelics in controlled, legal settings. We explore the evidence base linking each of the four types of STE to ecological wellbeing before proposing potential hypotheses to be tested to understand why STEs can have such beneficial effects. We end by looking at the factors that might need to be considered if STEs are going to be practically implemented as a means of achieving ecological wellbeing.

## Introduction

Ecological wellbeing describes the wellbeing of the planet and its inhabitants (Grouzet and Lee, 2014). The term recognizes the importance of the wellbeing of the earth’s natural systems alongside the wellbeing of humans and other species. Ecological wellbeing therefore incorporates sustainable resource use that avoids depleting natural systems beyond the planet’s “safe operating space” (Rockström et al., 2009) in addition to opportunities for human beings to experience hedonic (happiness and positive feelings) and eudemonic (having meaning and purpose) wellbeing (Ryan and Deci, 2001). Ecological wellbeing resonates with the responsive quality described by Martha Nussbaum when she outlined a central human capability to “live with concern for and in relation to animals, plants and the world of nature” that allows us to live a prosperous life (Nussbaum, 2011: 34). The achievement of ecological wellbeing is an important policy goal in line with international targets such as the Paris Climate Agreement (UNFCCC, 2015) and UN Sustainable Development Goals, designed to “shift the world on to a sustainable and resilient path” (Un General Assembly, 2015, p. 3).

In contrast, modern lifestyles are increasingly characterized by features that can work to undermine ecological wellbeing. These include unsustainable levels of consumption, strong materialistic values, and a disconnection between humans and nature (Kasser et al., 2004; Oliver, 2020; Jackson, 2021). Existing attempts to promote ecological wellbeing have mainly applied targeted interventions such as the provision of information (Abrahamse et al., 2007), activation of values (Perlaviciute and Steg, 2015), making pro-environmental identities more salient (Udall et al., 2020), or through the provision of far-reaching lifestyle change support mechanisms (Elf et al., 2019, 2021), among others. While these interventions have shown promising insights, they have *not* resulted in the necessary carbon reductions required to limit the increase in global average temperature to below 1.5°C above pre-industrial levels (UNFCCC, 2015). Exploration of further types of intervention is therefore needed. In this paper, we will explore an alternative type of potential intervention focused on the promotion of certain mental states. We will argue that there are specific types of mental states that may enhance our personal and collective wellbeing whilst also fostering a deep connection to and care for the environment. In this way, such mental states can operate as pathways toward ecological wellbeing.

The mental states that we are concerned with in this paper are self-transcendent experiences (STEs). STEs cover a class of mental states whereby the subjective experience of an independent self is temporarily reduced and feelings of connection to larger groups or entities are increased. STEs can occur naturally, through sometimes unexpected encounters with nature or during every-day activities that one intrinsically enjoys and lead to flow states but also through more intentional practices such as mindfulness and during therapeutic or ritualistic experiences with psychedelic substances such as LSD, psilocybin or Ayahuasca that can operate as a means to reach STEs.

In this paper, we explore how STEs can foster ecological wellbeing by examining the links between different types of STEs and both wellbeing and sustainability related variables. The paper is organized as follows. Section “The ills of modernity and their implications for ecological wellbeing” sets out the problems presented by modern lifestyles and why we need ecological wellbeing. Section “An introduction to self-transcendent experiences” then introduces STEs, exploring their characteristics and some of the different ways in which they can be experienced. Section “Varieties of self-transcendent experiences and their links to ecological wellbeing” is dedicated to examining each of four different varieties of STEs—awe (an emotional response to a conceptually or perceptually vast stimulus), mindfulness (a state of focused attention on the present moment), flow (a state of total immersion in an activity), and psychedelic experiences (changes in perception and emotion induced by the consumption of psychedelic substances such as LSD, psilocybin or Ayahuasca)—and their potential implications for ecological wellbeing. Section “Why do self-transcendent experiences support ecological wellbeing? Hypotheses to be tested” lays out our initial theoretical reasoning behind why STEs might support ecological wellbeing, proposing specific hypotheses to be tested. We finish in Section “Discussion: Practical considerations for using self-transcendent experiences as a means of intervening to promote ecological wellbeing” by discussing the factors that need to be considered if practically implementing intervention projects to use STEs as promoters of ecological wellbeing.

## The ills of modernity and their implications for ecological wellbeing

Over the course of the last two centuries, “the good life,” or what it means to live well, has increasingly been viewed as tightly linked to material wealth. Consumer advertising continues to tell us that we will be happier if only we could purchase the latest products. This materialistic view of the good life whereby “the more, the better” helps to promote the consumption behaviors that underpin consumer capitalist systems and support the growth paradigm (Fitzmaurice and Comegys, 2006; Kasser, 2017).

However, rising levels of consumption incur patterns of resource extraction that now threaten the health of the natural environment (Steffen et al., 2015a), pushing the earth’s support systems toward cataclysmic tipping points and beyond their carrying capacities (Steffen et al., 2015b). Whereas proponents of technological fixes have maintained that an ‘absolute decoupling’ between economic activity and environmental impact is possible, little evidence supports this appealing vision (Ward et al., 2016).

Further, the notion that consumption and the tightly linked idea of constant economic growth can deliver personal wellbeing has been shown to be tenacious alongside mounting scientific evidence documenting rising levels of distress in advanced economies (Blanchflower and Oswald, 2020), notwithstanding additional increases in gross domestic product (GDP). Empirical evidence documents that recent years have seen increasing numbers of people with depression^1^ and growing suicide rates (Winerman, 2019; Windsor-Shellard and Clay, 2020). Together with drug overdose and alcohol related liver diseases, these pose what Case and Deaton (2015) have come to call ‘deaths of despair.’

Another consequence of the inherent processes of modernity is the reduced feelings of connectedness between both people and their environment. French philosopher and writer Albert Camus suggested that the basic experience of modernity is one marked by hostility between human beings and the world (Camus, 1991: 14). When our need for connection and belonging is severely thwarted, we tend to care much more about status and popularity (Lavigne et al., 2011). A frequent response to the experience of loneliness is to try to numb the unpleasant feelings and emotions through the consumption of material goods as well as alcohol and other (recreational) drugs (Pieters, 2013; McKay et al., 2017). While these responses provide temporary distractions, with repeated exposure they lose their positive phenomenological effects, a process known as ‘neuroadaptation’ (Lembke, 2021: 53), and require ever greater dosages with potentially detrimental impacts on health and wellbeing.

Accordingly, modern lifestyles present problems for both the health of the planet as well as its inhabitants. In recognition of this, various intervention strategies have been proposed to encourage ecological wellbeing within modern societies. Many of these focus on telling individuals why they should care about the environment. For example, recent research is showing that highlighting the combined health/wellbeing and environmental benefits of sustainable actions such as reducing excess meat consumption can encourage engagement in those actions (Wolstenholme et al., 2020). Although these existing interventions show promise and are valuable, we are still far from achieving global sustainability goals. Achieving greater levels of ecological wellbeing therefore requires an exploration of further, new interventions that allow for wider shifts in thinking and acting away from currently detrimental states. In this paper, we will explore how STEs can act as a tool and potentially inform new interventions to facilitate this shift in mindset toward ecological wellbeing.

## An introduction to self-transcendent experiences

When we talk about a sense of self we are referring to the feeling that there is an ‘I’ or ‘me’ inside our heads who observes, interprets, and instigates actions in the world (Hood, 2012). This self is enduring and, in Western nations in particular, is also often experienced as independent and separate from others and the surrounding world (Cross et al., 2010; Santos et al., 2017). When asked “Who are you?”, people tend to answer with their unique personal characteristics, traits, abilities or likes.

During a STE, people’s sense of their independent self is temporarily altered. We adopt Yaden et al. (2017) conceptualization of STEs as encompassing two complementary components. The first involves a reduction in the salience of the boundaries between the self and ‘other’ alongside less pre-occupation with the self. In a self-transcendent state, people do not experience themselves as an isolated entity (sometimes described as ‘self-loss’) and are less self-focused. Instead, they introspect minimally and pay little attention to other people’s evaluations of them (Leary and Terry, 2012; Leary and Diebels, 2017). This component of STEs is sometimes referred to as ego-dissolution (Nour et al., 2017). The second, related component involves an increased sense of connectedness. This can be with other people, objects, nature or even the cosmos. According to Kaufman (2020: xxxiv), self-transcendence allows for the “highest levels of unity and harmony within oneself and with the world.” At its most extreme, during STEs, people may experience themselves as what D’Aquili and Newberg (1999) describe as an ‘‘Absolute Unitary Being.’’^2^

The term ‘self-transcendence’ has been used widely across different disciplines (Kitson et al., 2020). Frankl (1966) used ‘self-transcendence’ to refer to the human capacity to be motivated by trying to help others rather than seeking pleasure for oneself whilst Cloninger et al. (1993) consider self-transcendence as a trait representing the extent to which people feel that they are part of the universe as a whole. Further, scholars such as Erikson (1959) and Maslow (1962) include self-transcendence in their theories of human development where it is often considered to represent the final or highest stage of human development. In all these cases, self-transcendence refers to longer-lasting states of being or changes in perspectives. These definitions do not fully align with our perspective as we are interested in single, transient self-transcendent *experiences* as a form of intervention. Whilst it may be the case that STEs can lead to changes in how people view the world, such changes in motives or behavior do not have to display themselves within the STEs itself as we define it.

Several different mental states can be considered to represent a STE (Yaden et al., 2017; Yaden and Newberg, 2022). These include mystical experiences that are often facilitated through religious practices, peak experiences, emotional responses such as sensations of awe and feelings of love and gratitude, mindfulness, flow, and the state of consciousness induced *via* psychedelic substances, amongst others. Across all these different states, individuals experience a degree of reduced self-focus and enhanced connectedness to larger entities. Some of these states are typically experienced as very intense such as experiences facilitated by psychedelic substances, which are sometimes employed within ritualistic practices (Arce and Winkelman, 2021; Dupuis, 2021) and have increasingly been tested as a form of therapy for various mental health conditions (Wolff et al., 2020; Villiger, 2022). Others are less intense and may be experienced by some individuals on a day-to-day basis. For example, during flow states individuals can lose themselves in activities such as reading a good book, playing sports, or doing arts and crafts. In line with this, STEs have been suggested to occur along a spectrum of intensity.

From this wider list we have chosen to focus our attention on four varieties of STE: awe, mindfulness, flow, and psychedelic-induced experiences. These states were selected because they are clearly differentiated from one another in the academic literature and emerging and existing evidence is already exploring their associations with variables relevant for ecological wellbeing. Psychedelic-induced experiences were chosen because of the resurgence of research interest in their potential benefits and neurological underpinnings over recent years in universities across the world, which is helping to develop our understandings of the nature of STEs. Our focus on psychedelic experiences is limited to academic research which has been conducted with ethical approval and whereby the nature and effects of psychedelic-induced experiences have been examined in controlled, legal settings. Together, these four varieties of STE cover a range of states that fall across the spectrum of intensity mentioned above whilst sharing similar a self-transcendent aspect.

## Varieties of self-transcendent experiences and their links to ecological wellbeing

This section of the paper is dedicated toward exploring four different varieties of STEs—awe, mindfulness, flow, and psychedelic-induced experiences along with how they may support ecological wellbeing. For each variety of STE, we introduce the concept and then outline the empirical evidence base surrounding its links to ecological wellbeing.

### Awe and ecological wellbeing

Awe is a complex emotion that is suggested to be triggered when an individual is faced with a stimulus that they perceive to be perceptually and/or conceptually vast and requires them to adjust their current mental schemas in order to accommodate this new information (Keltner and Haidt, 2003). It often includes heightened feelings of wonder, appreciation, joy, and inspiration as well as fear and a sense of being overwhelmed (Yaden et al., 2019).

Common elicitors of awe include nature scenes, art, music, extraordinary talent, religious experiences and the birth of a child (Shiota et al., 2007; van Elk et al., 2016; Chirico and Yaden, 2018). Awe can also be experienced through more mundane, shared, and unifying experiences such as during demonstrations or a concert, which led David Yaden to refer to awe as “the everyperson’s spiritual experience” (Kaufman, 2020: 206). It is important to note that awe can be prompted by both positive (e.g., natural wonders) and negative (e.g., natural disasters) stimuli (Gordon et al., 2017) and the nature of the associated awe experience can differ based on this. For example, awe prompted by beautiful scenery may be characterized by feelings of surprise, wonder and humility whilst awe prompted by a natural disaster (or a worldwide pandemic) may be characterized by feelings of fear, disorientation and uncertainty (van Elk et al., 2016).

When experiencing awe, an individual is suggested to feel small and insignificant in relation to the vast and overwhelming stimuli they are confronted with (Campos et al., 2013; Piff et al., 2015; Bai et al., 2017). This leads them to direct their attention away from themselves (Shiota et al., 2007) and widens their attentional focus toward entities that are larger than the self (Sung and Yih, 2015). In this way, awe encapsulates the ego-dissolution aspect of STEs because self-referential processing is reduced. At the same time, this reduction in the salience of the self during awe is accompanied by feelings that one belongs to a larger or universal group such as their neighborhood, nation or species (Shiota et al., 2007; Van Cappellen and Saroglou, 2012; Chen and Mongrain, 2020), thus encompassing the increased feeling of connection aspect of STEs.

Following the publication of Keltner and Haidt’s (2003) seminal paper, numerous studies have linked awe with higher human wellbeing. For example, awe has been associated with greater levels of life satisfaction (Rudd et al., 2012; Krause and Hayward, 2015), more positive emotions (Anderson et al., 2018; Rankin et al., 2019), and increases in meaning in life (Rivera et al., 2019). Awe might also help to moderate the effects of stress on the body (Chen and Mongrain, 2020) in that it is associated with increased activation of the parasympathetic branch of the autonomic nervous system (ANS) (Chirico et al., 2017) and reduced activation of the sympathetic branch of the ANS (Shiota et al., 2011).

The experience of awe has been positively linked to several factors related to sustainable behaviors (Zelenski and Desrochers, 2021), thus contributing to ecological wellbeing. For example, induced awe experiences can lead to greater intentions to engage in resource saving behaviors such as recycling or taking shorter showers (Yang et al., 2018; Bethelmy and Corraliza, 2019), reduced preferences for material goods and conspicuous consumption (Rudd et al., 2012; Hu et al., 2018), as well as a greater likelihood of purchasing of ‘green’ products (Wang et al., 2019). Following experiences of awe, individuals are more willing to sacrifice their own standard of living in order to protect the environment (Zhao et al., 2018) and donate greater amounts of money to environmental charities (Ibanez et al., 2017). Research has shown that awe can increase feelings of connectedness to nature (Nelson-Coffey et al., 2019), which has been shown to mediate the relationship between awe and engagement in pro-environmental behavior (Yang et al., 2018; Sun et al., 2021).

### Mindfulness and ecological wellbeing

Mindfulness describes a state of being whereby attention is focused on the present moment (Kabat-Zinn, 1994). A mindful individual aims to observe their mental states and outside events as they happen, on a moment-to-moment basis but does not react to them in an automatic or emotionally charged way (Bishop et al., 2004). Rather, mindfulness practitioners observe their thoughts and experiences in a non-judgmental manner, and more consciously choose their reactions to these (Chambers et al., 2009). By focusing on the present moment, mindful individuals are not distracted by ruminations about the past or hopes and anxieties about the future (Armstrong and Jackson, 2015). Mindfulness can be cultivated through the practice of meditation whereby individuals purposefully self-regulate their attention (Baer, 2003), for example to focus on a selected sound (Lynch et al., 2018), object, or bodily sensation (Lee et al., 2012).

A number of researchers have explicitly stated that mindfulness involves a self-transcendent element (Garland and Fredrickson, 2019). Vago and Silbersweig’s (2012) S-ART framework proposes that mindfulness can develop a type of relationship between self and other that transcends self-focused needs and increases prosocial tendencies. Hanley et al. (2020) outline how mindfully attending to specific objects decreases self-referential thoughts. The process of becoming an objective observer of one’s own stream of consciousness, sometimes referred to as ‘decentering,’ can also reduce the extent to which people identify with their existing, static sense of self (Hölzel et al., 2011). Research has shown that taking part in a mindfulness training session can lead to the dissolution of body boundaries (the extent to which the self is experienced as discrete and separate from the surrounding world) (Dambrun, 2016; Nave et al., 2021) and promote an allocentric rather than egocentric frame of reference (Hanley and Garland, 2019). An allocentric frame of reference denotes feelings of unity with the social and natural worlds whilst an egocentric frame of reference denotes a self-centered preoccupation with internal events (Hanley et al., 2020).

The human wellbeing benefits of practicing mindfulness are well documented. On the one hand, mindfulness can improve outcomes for clinical patients experiencing problems such as anxiety (Kabat-Zinn et al., 1992) and cancer (Speca et al., 2000). On the other hand, practicing mindfulness is associated with reductions in stress (Astin, 1997) and symptoms of depressions (Shapiro et al., 1998), alongside greater life satisfaction (Crego et al., 2019), positive feelings more generally (McKay and Walker, 2021), and higher self-esteem (Pepping et al., 2013) in non-clinical populations.

With regards to environmental sustainability, numerous studies have shown that people who are more mindful tend to engage in a greater number of ecologically sustainable behaviors (Brown and Kasser, 2005; Ericson et al., 2014; Wamsler et al., 2018). Amel et al. (2009) found that individuals who were more mindful tended to state that they chose to engage in the most sustainable option available to them more often than those who were less mindful. Similarly, Panno et al. (2018) reported that people higher in trait levels of mindfulness had a stronger tendency to engage in behaviors such as saving energy and water, recycling and having a more sustainable diet. Again, it appears that increased connectedness to nature could partly account for why mindfulness is positively linked to pro-environmental behavior (Barbaro and Pickett, 2016). In addition, Rosenberg (2004) argued that mindfulness could serve as an antidote to consumerism in that it enables people to be more aware of their automatic responses to consumer advertising, leading them to be less likely to make impulsive purchases. This combination of positive effects on human wellbeing alongside an increase in ecologically sustainable behaviors indicates that mindfulness has the potential to support ecological wellbeing.

### Flow experiences and ecological wellbeing

Flow describes a state of optimal experience whereby an individual is totally immersed in what she or he is doing. It is about the intense absorption in an activity that results in individuals thinking about nothing else in the moment (Jackson and Eklund, 2004). The flow concept was first identified by the psychologist Csikszentmihalyi (1975) while conducting interviews with musicians, artists, rock-climbers, and surgeons, amongst others. Csikszentmihalyi found that in all these different activities, people were reporting similar experiences of total absorption.

Flow experiences are typically considered to involve nine components or characteristics. These outline the different conditions that are usually present and feelings that people have when they are in a flow state. The conditions that support flow include a perceived balance between the skills that a challenge requires and those that an individual possesses (Csikszentmihalyi, 1975; Moneta and Csikszentmihalyi, 1996), that the activity has clear goals, and that the individual is provided with immediate feedback concerning their progress toward these (Csikszentmihalyi, 1990; Jackson and Eklund, 2004).

During flow, individuals devote all their attention to the activity they are engaged in, creating an experience of total concentration. Further, as no attention is granted to anything other than the activity, this prevents an individual from perceiving themselves as a separate entity from the actions they are performing (Csikszentmihalyi, 1992). There is no attention left to attend to the self and as such individuals’ actions feel spontaneous or effortless because they are not aware of any conscious effort to initiate them. For similar reasons, during flow all self-consciousness disappears and therefore, rather than being pre-occupied with living up to a certain standard, individuals show reduced concerns with what other people may be thinking about them (Csikszentmihalyi, 1990). This blurring of the boundary between self and activity and loss of self-consciousness is what makes flow a STE.

Due to the extreme immersion experienced during flow, individuals’ perception of time is altered, most commonly such that hours seem to go by in minutes. Individuals also experience a sense of control; that they are acting freely and can directly influence the outcome of the activity. The flow experience is also intrinsically motivating. That is, it is enjoyable and rewarding so that individuals will choose to engage in the respective activity simply for the sake of experiencing flow, rather than for any external rewards (e.g., money or praise) it may bring (Csikszentmihalyi, 1992; Jackson and Eklund, 2004).

On top of being inherently enjoyable, frequent flow experiences have been linked to enhanced individual wellbeing. Research shows that people who experience flow more often also tend to have greater self-confidence (Hektner and Csikszentmihalyi, 1996), higher life satisfaction (Bryce and Haworth, 2002; Tse et al., 2020) and a stronger sense of fulfilment (Asakawa, 2004). A single, high quality experience of flow can also give people a boost in positive feelings immediately after the activity (Mundell, 2000; Fullagar and Kelloway, 2009; Rogatko, 2009). Indeed, flow has been outlined as a core element that can deliver psychological wellbeing in theories from Positive Psychology (Seligman, 2012).

For the previous types of STE that we have explored, there is evidence to suggest that the STE can lead to increases in pro-ecological values and/or behaviors. Emerging, unpublished findings are starting to document that frequent experiences of flow predict increases in self-transcendent values such as universalism, which encompasses care for all living things (Isham and Jackson, 2022). Empirical evidence also documents that flow experiences are more likely to occur in activities with lower environmental costs - as measured by greenhouse gas intensity (Isham et al., 2019). In particular, flow seemed to be occurring in activities such as talking with family and friends, prayer, yoga, arts and crafts, singing and dancing, cycling and aerobics. None of these necessitate unsustainable amounts of physical energy and materials. In this way, by providing satisfaction through engagement in less environmentally costly activities, flow experiences may be able to support human wellbeing without compromising the wellbeing of the environment.

### Psychedelic-induced experiences and ecological wellbeing

Drawing on the Greek words “psyche” (for mind or soul) and “deloun” (for show), the term ‘psychedelic’ is now often translated as “mind-manifesting” (Dyck, 2006). Psychedelic substances are broadly divided into *classic* psychedelics and *non-classic* psychedelics. The former provides a category of psychedelics which includes, among others, psilocybin, Ayahuasca and LSD. In this paper we will focus on classic psychedelics, which have been defined as “[a] drug which, without causing physical addiction, craving, major physiological disturbances, delirium, disorientation, or amnesia, more or less reliably produces thought, mood, and perceptual changes otherwise rarely experienced except in dreams, contemplative and religious exaltation, flashes of vivid involuntary memory, and acute psychosis” (Grinspoon and Bakalar, 1979: 9).

Despite reviews showing psychedelics relative safety and tolerability (dos Santos et al., 2018; Schlag et al., 2022), they remain Schedule I drugs and are still illegal in most countries with a few exceptions. In addition, there are some significant risks associated with the use of psychedelics, particularly for certain ‘at risk’ groups, which we will consider in more detail in Section “Social acceptability of the different approaches.” We therefore wish to make clear that we are not advocating for the uncontrolled consumption of substances that are currently illegal in order to achieve sustainable wellbeing. However, given that there has been a boom in research interest in the nature and effects of medically supervised psychedelic-induced experiences in recent years, alongside the fact that they represent an often highly intense form of STE, the supervised, legal use of psychedelics is still important to consider for our investigation. There are now numerous clinical trials of psychedelic-assisted therapy whereby psychedelic substances are administered legally under professional provision in controlled environments. Recent research also suggests that medical supervision can help to alleviate many of the risks of psychedelics as safe use guidelines become increasingly well-defined (Schlag et al., 2022). Alongside this, there are calls for a ‘behavioral psychedelics’ (Neuhaus and Slavich, 2022), whereby psychedelics could be used as a means of fostering positive health behaviors and resilience. Should such forms of psychedelic therapy become more widespread in the future, then these controlled settings may offer specific situations whereby psychedelics could be safely and effectively administered to support desirable outcomes.

Classic psychedelics often occasion extreme changes in one’s mental state (Passie et al., 2002; Griffiths et al., 2006, 2011). During a psychedelic-induced state, people can experience alterations in their visual and psychological perceptions (Huxley, 1954). They may see different colors and shapes or sense a change in their mood or perceptions. Most importantly, for our purposes, a psychedelic-induced state is often characterized by feelings of oneness, that all things are interconnected, and ego-dissolution; that the border between one’s self and the outside world is breaking down (Blatchford et al., 2021). In this way, psychedelic experiences can be considered as self-transcendent.

Classic psychedelics contain psychoactive compounds that exercise their effects through agonist (including partial agonist) activity at the serotonin 2A receptor (5-HT_2*A*_R; Tagliazucchi, 2020). Studies on human propositi by Kometer et al. (2012, 2013); Quednow et al. (2012) have shown that 5-HT_2*A*_R antagonism blocks the subjective and other neurological effects of the classic psychedelic psilocybin. Despite the primary role of 5-HT_2*A*_R agonism, other receptor-level mechanisms such as 5-HT_2*C*_ and 5-HT_1*A*_ receptors are associated with psychedelic effects (Halberstadt and Geyer, 2011) in the form of STEs. Higher doses of psychedelic substances trigger strong STEs (Millière et al., 2018) and are typically employed in psychedelic therapy (Garcia-Romeu and Richards, 2018; Nutt and Carhart-Harris, 2021).

Growing research interest on psychedelics has led to an accumulation of evidence in recent years showing their potential to actively support human health outcomes (dos Santos et al., 2016; Johnson and Griffiths, 2017; Johnson et al., 2019; Raison et al., 2022). Psychedelic substances have been shown to have treatment potential for physical health problems such as alcoholism (Hoffer, 1967; Dyck, 2006; Bogenschutz et al., 2015) and cluster headaches (Sewell et al., 2006) as well as mental health problems such as depression, anxiety, addiction, obsessive-compulsive disorder and anorexia (Bogenschutz et al., 2015; dos Santos et al., 2016; Patra, 2016; Ross et al., 2016; Sanches et al., 2016; Roseman et al., 2018), among others. Notably, this field is still in its infancy and more research is required to consolidate findings. For instance, whereas small-scale open-label trials suggested, in the case of depression, that psilocybin has more positive results than traditional antidepressant treatments (Erritzoe et al., 2018), more recent findings show no superiority of psychedelics over traditional antidepressants in terms of treatment efficacy (Carhart-Harris et al., 2021). However, what remains is that psychedelic therapy provides a good alternative for those patients appearing treatment-resistant to traditional antidepressants.

Self-transcendent experiences induced through clinically regulated use of psychedelics can also support non-clinical aspects of human wellbeing (Mans et al., 2021). For example, psilocybin has shown to reduce fear of death, while, simultaneously, increasing a person’s sense of purpose and having enduring positive impacts on mood (Yaden et al., 2016; Barrett and Griffiths, 2018). The human wellbeing effects of psychedelic experiences appear to be long-lasting with studies documenting increases in life satisfaction, meaning in life and reduced stress sustained at 4-weeks to 14-months follow-ups (Griffiths et al., 2011; Schmid and Liechti, 2018; Uthaug et al., 2018).

Psychedelic-induced STEs have also been linked to increases in care for the environment and engagement in pro-environmental behaviors, thus demonstrating their potential to support ecological wellbeing. Numerous studies have documented that the use of psychedelics has been linked to increases in nature relatedness (Nour et al., 2017; Lyons and Carhart-Harris, 2018; Kettner et al., 2019; Nayak and Griffiths, 2022). As with awe experiences, these increases in feelings of connectedness to the natural world have been shown to account for why psychedelic-induced experiences have also been linked to increases in engagement in pro-environmental behaviors such as recycling and trying to conserve water (Forstmann and Sagioglou, 2017; Whitburn et al., 2020).

Drawing on the analogy of astronauts when experiencing what has come to be known as the ‘overview effect’ (see also Yaden et al., 2016), psychedelic-induced STEs have been shown to incur a greater appreciation of *Earth* as a common place shared by all of humanity, thus potentially fostering a sense of ‘global citizenship.’ This awareness can lead to an improved ecological sensitivity and, consequently, a greater commitment to protect nature. This is perhaps best captured in a quote from a research participant in a study conducted by Lyons and Carhart-Harris (2018: 817):“Before I enjoyed nature, now I feel part of it. Before I was looking at it as a thing, like TV or a painting… [But now I see] there’s no separation or distinction, you are it.”

## Why do self-transcendent experiences support ecological wellbeing? Hypotheses to be tested

Thus far we have seen that the four different varieties of STE all, if appropriately implemented, have the potential to support ecological wellbeing in the sense that they have been shown to be positively linked to, or able to enhance, human health and wellbeing alongside pro-environmental values and behaviors. In this section, we consider *why* STEs have these common consequences. Is there something inherent within the nature of STEs that directly impacts ecological wellbeing? Or do they all exert their effects through a further, common mediating variable? We propose several possible explanatory factors that could be explored, empirically, in future work.

### Lasting changes in self-construal

The defining characteristics of STEs state that individuals experience an alteration in their sense of self such that it is less independent and more connected to the world around them. This expansion of one’s self-concept to include, perhaps, other people and the environment could account for why STEs support ecological wellbeing, especially if the alterations to self-concept last beyond the STE itself. Indeed, many scholars have argued for the value in expanding from an ‘egoic’ to an ‘ecological’ self (Naess, 2010). For example, in his book, The Righteous Mind, social-psychologist Jonathan Haidt suggests that “We may spend most of our waking hours advancing our own interests, but we all have the capacity to transcend self-interest and become simply a part of a whole. It’s not just a capacity; it’s the portal to many of life’s most cherished experiences” (Haidt, 2013).

Given that ecological behaviors often involve the sacrifice of individual interests in exchange for the overall interests of people and nature (Yang et al., 2018), those individuals whose sense of self has been expanded to include other people, one’s environment or even the world at large (Aron et al., 2000; Leary et al., 2008) should be more likely to care for the environment (i.e., because it is not distinct from themselves). In support of this, individualistic societies, whereby the independent self is dominant, have been shown to have higher ecological footprints (Komatsu et al., 2019). Further, an independent self-construal (or representation of the self) has been linked to only showing concern for environmental degradation when the consequences will have a direct, negative impact on the self, rather than because the environment is intrinsically valuable (Davis and Stroink, 2016). The expansion of the self to include other people could also help to remedy what Cushman (1990) described as an “empty self,” whereby people feel they must fill the self with consumer products in order to fill the void caused by a lack of community and shared meaning. If the self is no longer ‘empty,’ people may feel less of a need to engage in non-essential consumption practices to fill it.

In addition, the expansion away from an overly individualistic sense of self could support human wellbeing. Clinical research documents that excessive focus on the individual self (especially its detrimental aspects) is linked to negative affect (Mor and Winquist, 2002), social anxiety (Spurr and Stopa, 2002) and depression (McLaughlin and Nolen-Hoeksema, 2011). Moreover, psychological research shows that the perception of the permanent and independent self is linked to fluctuating rather than authentic-durable happiness (Dambrun and Ricard, 2011; Dambrun, 2017).

A first set of hypotheses to be tested is therefore whether STEs lead to lasting changes in one’s self-construal and whether such changes in self-construal mediate the positive associations between the different varieties of STE and ecological wellbeing.

### Neurological triggers

The exploration of the neurological correlates of the different varieties of STEs outlined in this article reveals a number of common neurological features. Here, we wish to focus on two such features. The first involves the activation of a large-scale brain network known as the Default Mode Network (DMN). The second involves an increase in dynamic activity across brain networks. Whilst it has been suggested that these two features may be related to the common phenomenological features of STEs, further research is needed to determine whether such patterns of neurological activation are required for the positive effects that STEs appear to have on ecological wellbeing to appear. In other words, research is needed to ascertain whether specific neurological markers such as altered activity within the DMN and increases in global functional connectivity need to be present to experience the positive consequences of STEs for ecological wellbeing.

The DMN comprises several brain regions that are active during rest, but less so during goal-directed engagement in tasks that require attention (Blatchford et al., 2021). Examples of some of the brain regions included within the DMN are the medial prefrontal cortex, medial and lateral parietal cortex, medial temporal lobes and posterior cingulate cortex (Andrews-Hanna et al., 2014). A key characteristic of the DMN is that its regions are highly interconnected and show high heteromodality (Carhart-Harris and Friston, 2010). The DMN is involved in a number of self-related thought processes. These include self-consciousness, self-referential thought, mind-wandering and rumination, whereby people repetitively focus on often negative thoughts related to themselves (Carhart-Harris and Friston, 2010; Blatchford et al., 2021). Carhart-Harris and Friston (2010) even speculated that the network could be the primary neurological basis of the Freudian ego.

Several studies have now documented that during STEs, individuals show an altered pattern of activity within the DMN. Most commonly, studies report a reduction in functional connectivity^3^ within the DMN^4^ (Millière et al., 2018) as well as reduced activity within the brain structures that form part of the DMN such as the posterior cingulate cortex (Muthukumaraswamy et al., 2013; Palhano-Fontes et al., 2015) and medial prefrontal cortex (Carhart-Harris et al., 2012). These effects have been found for STEs occurring under LSD (Carhart-Harris et al., 2016b), psilocybin (Carhart-Harris et al., 2012), and Ayahuasca (Palhano-Fontes et al., 2015), as well as those occurring during awe (Guan et al., 2018; van Elk et al., 2019), mindfulness (Farb et al., 2007; Brewer et al., 2011; Garrison et al., 2014; Berkovich-Ohana et al., 2015; Tang et al., 2015; Scheibner et al., 2017; Lin et al., 2018), and flow (Ulrich et al., 2014, 2016, 2018; Keller, 2016). Reduction in activity within the DMN has been correlated with the subjective experience of ego-dissolution (Muthukumaraswamy et al., 2013; Carhart-Harris et al., 2016b).

Alongside a reduction in activity within the DMN, STEs also appear to be linked to an *increase* in spontaneous and dynamic brain activity, especially among high-level association networks (Schartner et al., 2017; Tagliazucchi, 2020; Blatchford et al., 2021). In a phenomenon sometimes called the ‘entropic brain’ in psychedelic research (Carhart-Harris et al., 2014; Herzog et al., 2020), brain activity becomes disorganized allowing for an increased connectivity between usually distinct brain networks. For example, Carhart-Harris et al. (2013) showed that the DMN and the Task-Positive Network (which serves to support focused attention), whose activities are usually negatively correlated, displayed greater functional connectivity under the effect of psychedelics. Again, such patterns of brain activity have been observed under various classic psychedelics in clinical studies including LSD (Müller et al., 2017), psilocybin (Roseman et al., 2014), and Ayahuasca (McKenna and Riba, 2018).

An increase in functional connectivity across different brain areas has also sometimes been documented during awe. For example, Takano and Nomura (2022) found that during awe experiences, the right anterior supra-marginal gyrus (aSMG) showed increased functional connectivity with brain structures such as the amygdala and Middle Temporal Gyrus (MTG). Likewise, mindful states have been linked to increased functional connectivity across usually distinct brain networks (Kral et al., 2019) such as the Central Executive Network (which is involved in sustained attention, working memory and decision making related to goal-directed behavior) and the DMN (Brewer et al., 2011; Bauer et al., 2019). The synchronization theory of flow (Weber et al., 2009) highlights that during a flow experience, an increased synchronization of activity takes place across brain networks involved in cognitive control and those involved in reward processing (Huskey et al., 2018). See Table 1 for a summary of research linking each of the four varieties of STE to these neurological patterns.

**TABLE 1:** Summary of common neurological features across four varieties of self-transcendent experience (STE).

Neurological correlate	Awe	Mindfulness	Flow	Psychedelic-induced STE
Reduced activation of DMN structures/reduced functional connectivity (FC) within the DMN	✓	✓	✓	✓
	Guan et al., 2018; van Elk et al., 2019	Farb et al., 2007; Brewer et al., 2011; Garrison et al., 2014; Lin et al., 2018	Ulrich et al., 2014, 2016, 2018; Berkovich-Ohana et al., 2015; Tang et al., 2015; Keller, 2016	Carhart-Harris et al., 2012, 2016b; Muthukumaraswamy et al., 2013; Palhano-Fontes et al., 2015
Increased functional connectivity (FC) across brain networks	✓	✓	✓	✓
	Between right anterior supra-marginal gyrus and brain structures such as the amygdala and middle temporal gyrus (Takano and Nomura, 2022)	Between dorsolateral prefrontal cortex and posterior cingulate cortex (Kral et al., 2019) Between DMN and Central Executive Network (Bauer et al., 2019).	Between brain networks involved in cognitive control (dorsolateral prefrontal cortex, thalamus) and reward processing (putamen) (Huskey et al., 2018).	Between DMN and Task-Positive Network under psilocybin (Carhart-Harris et al., 2013) Between thalamus and right fusiform gyrus and insula under LSD (Müller et al., 2017) Between numerous networks including DMN, Visual-Lateral Network, and Executive Control Network under psilocybin (Roseman et al., 2014) Between posterior and anterior brain regions under Ayahuasca (McKenna and Riba, 2018)

These increases in spontaneous neural activity and global connectivity have been shown to correlate with subjective reports of ego-dissolution (Nour et al., 2016; Tagliazucchi et al., 2016; Schartner et al., 2017) and changes in perceived body boundaries (Hanley et al., 2020). Carhart-Harris et al. (2017) speculated that this increase in global functional connectivity could be the neural correlate of the unitive feelings of connectedness during psychedelic-induced STEs. Further, neuroscientists believe that this freed and unimpeded communication across brain networks holds profound therapeutic potential. Carhart-Harris succinctly compared this effect to a mountain with usually well-trodden paths or slopes that, after the consumption of psilocybin, resembles a mountain with fresh snow, thus allowing the brain to engage in new ways of operating (Illing, 2019). This pattern of brain activity may therefore account for why mindfulness has been proposed as a way of overcoming the “polarized mind” (Schneider, 2013), that is, the fixation on a single point of view while excluding competing points of view.

Future research could therefore usefully test whether (a) reduced activity within the DMN and (b) increases in global functional connectivity are a necessary determinant of the positive effects of STEs on ecological wellbeing. These two neural signatures represent what we feel are, as of today, the most well-documented patterns of neural activation across the different varieties of STE. Yet, there is still much to learn about the neuroscience of STEs. There might be other brain areas involved in facilitating different varieties of STEs that have not yet been fully explored. For example, the experience of awe has been negatively correlated with regional gray matter volume in the insula (Guan et al., 2019) - a brain area implicated in interoception and the experience of bodily self-awareness (Zaki et al., 2012). Future research could test if this neurological feature is also present during psychedelic experiences, mindfulness, and flow.

The work of Colombia psychologist Lisa Miller on religion and spirituality (Miller et al., 2012) may also offer some clues to other brain areas potentially involved in facilitating STEs. According to Miller (2021: 161), during a spiritual experience, hard, fixed boundaries soften, a feeling of separateness is weakened, and we tend to “embrace sensations of transcendence and union,” thus sharing significant similarities with STEs’ effects explored in this paper. Recent research from her lab has found that during a spiritual experience greater activation in the ventral attention network and the frontotemporal network can be observed as well as reduced activation in the inferior parietal lobe, where we navigate perceived distinctions between self and others (McClintock et al., 2019). Such brain areas may also have relevance for the different varieties of STE that we have explored in this paper and could be explored in future work.

### Increases in empathy

A further means through which STEs may support ecological wellbeing is by increasing empathy. Empathy describes the ability to imagine oneself in the circumstances of another person and be able to understand how they may be feeling. Once people are able to appreciate how their behavior may be affecting other people, species and future generations, they seem to become motivated to behave in more pro-social and pro-environmental ways (Berenguer, 2007; Lu and Schuldt, 2016; Brethel-Haurwitz et al., 2020). It is clear then, how empathy could theoretically explain the positive effects of STEs on planetary health. Likewise, a number of psychological studies have documented positive relations between individuals’ level of empathy and their personal wellbeing (Morelli et al., 2015; Vinayak and Judge, 2018).

Notably, evidence already documents that mindfulness can foster empathy (Shapiro et al., 1998; Jones et al., 2019) as can psychedelic-induced STEs (Dolder et al., 2016; Pokorny et al., 2017; Blatchford et al., 2021). When assessing the impact of psilocybin consumed in a retreat setting, Mason et al. (2019) found that individuals displayed higher empathy the morning after psilocybin consumption and this effect was retained seven days later. In contrast, less work has been conducted to explore how the experience of awe and flow might lead to changes in levels of empathy. However, the right aSMG, which has been documented to show increased functional connectivity during awe experiences, has also been linked to empathetic responses (Miller et al., 2020).

The reduction in the salience of the boundary between self and other during STEs may encourage the increases in empathy. By reducing the extent to which people consider themselves as separate from other people, this may increase the ability to empathize with others (Schmid and Liechti, 2018). In this way, the alterations in an individual’s self-construal during STEs may encourage the capacity for empathy and, as such, these two proposed mediating variables may operate together to account for why STEs support ecological wellbeing. Further hypotheses to be tested therefore include whether increased empathy mediates the relationship between the different varieties of STEs and ecological wellbeing, and how self-construal and empathy may work together to mediate the relationship between STEs and ecological wellbeing.

### Changes in personality

A final way in which we wish to propose that STEs may support ecological wellbeing is by encouraging the personality trait of ‘openness’ (Goldberg, 1993). Openness has been linked to outcomes that are beneficial for planetary health. Individuals who are more open to new experiences and ideas are likely to be more willing to change and transform their lifestyles in favor of those that benefit the environment. Multiple studies now document a positive association between the extent to which an individual is high on openness and their environmental concern and engagement in pro-environmental behaviors (Hirsh, 2010, 2014; Markowitz et al., 2012; Milfont and Sibley, 2012). Some research also documents that openness is a trait that predicts higher levels of psychological wellbeing (Kokko et al., 2013).

Existing research links each of the four varieties of STEs that we have discussed in this paper with higher levels of openness. Individuals shown to be prone to experience sensations of awe have been documented to report higher levels of openness (Nusbaum and Silvia, 2010; Stellar et al., 2015) whilst those high in need for cognitive closure (a desire to avoid ambiguity) are less awe-prone (Shiota et al., 2007; Pilgrim et al., 2017). Openness has also been suggested to be positively related to an individual’s tendency to experience flow (Baumann, 2012). A meta-analysis between mindfulness and personality traits found a positive correlation between the extent to which individuals were mindful and the trait of openness (Giluk, 2009) whilst other research has shown that following a brief period of mindfulness training, participants tend to report higher openness to experience scores (Chan et al., 2019). Scores on measures of this personality trait have been documented to increase following a psilocybin or LSD session in clinical psychedelic research (MacLean et al., 2011; Carhart-Harris et al., 2016a; Erritzoe et al., 2018).

A final set of hypotheses to be tested therefore revolve around whether higher levels of openness mediate the relationship between STEs and ecological wellbeing. Part of this research will have to involve determining whether the different STEs cause changes in openness, rather than just reporting simple correlations.

## Discussion: Practical considerations for using self-transcendent experiences as a means of intervening to promote ecological wellbeing

In this paper, we aimed to explore the potential of STEs for the achievement of ecological wellbeing in an attempt to tackle the ills of modernity. In Section “Varieties of self-transcendent experiences and their links to ecological wellbeing,” we saw how STEs induced through awe, mindfulness, flow, and the regulated intake of some psychedelic substances have all been linked to higher levels of environmental concern and/or more sustainable behaviors, alongside high levels of human wellbeing. Section “Why do self-transcendent experiences support ecological wellbeing? Hypotheses to be tested” highlighted multiple hypotheses to be tested surrounding *why* STEs can have beneficial effects on ecological wellbeing. If we are to accept the proposal that STEs offer a valuable route toward achieving greater ecological wellbeing, a subsequent question might be how can we encourage engagement in STEs by individuals? It is rather unlikely that everyone can suddenly start to experience STEs without any change in our current mindsets or lifestyles. We will need interventions designed to increase the accessibility of STEs in order to meet environmental targets. As explored in this paper, these could be through making mindfulness training freely available, through opportunities to visit awe-inducing sites, or through ‘social prescribing’ (Buck and Ewbank, 2020) to refer people to engagement in flow-conducive activities, for example. Whilst we are hesitant to make specific recommendations concerning the exact contents or format of interventions until the hypotheses outlined in Section “Why do self-transcendent experiences support ecological wellbeing? Hypotheses to be tested” have been sufficiently addressed, we will use this final section to consider points relevant for how we can practically begin to use STEs to promote ecological wellbeing. Here, we discuss whether different varieties of STE could and/or should be promoted together or independently, how frequently STEs need to happen to produce sustained changes in wellbeing and pro-environmental attitudes and behaviors, their differing levels of social acceptability, and how their effectiveness may be impacted by motives that are consistent with consumer capitalism.

### Independent or combined approaches?

In recent years, several studies have started to explore how different varieties of STEs relate to each other beyond their shared self-transcendent aspects. In this way, rather than interventions trying to promote either one type of STE or another, it may be more desirable to design interventions that can promote multiple varieties of STEs concurrently. Such approaches may also be able to amplify the positive effects of each type of STE involved in the intervention.

As an example, mindfulness could be used to increase people’s propensity to experience flow. In sports settings, mindfulness training has been shown to increase athletes’ likelihood of experiencing flow (Aherne et al., 2011). Pizarro et al. (2020) also documented that a mindful-dancing program was able to have positive effects on participants levels of compassion (a positive determinant of pro-environmental attitudes, Lu and Schuldt, 2016) and that this effect was mediated by high levels of shared flow experiences amongst the participants. Accordingly, we can see that mindfulness may be able to support the experience of flow, and then when both states operate together, they can have beneficial effects for ecological wellbeing.

As another example, it has been suggested that combining psychedelic and mindfulness interventions could lead to greater beneficial effects on mental health and wellbeing (Payne et al., 2021). Smigielski et al. (2019) found that administering psilocybin to practitioners during a mindfulness meditation retreat led to increased meditation depth and more profound, positive experiences of ego-dissolution. At 4-month follow-up, meditators who had also taken psilocybin at the retreat reported significantly greater appreciation of life, self-acceptance, quest for meaning and sense of purpose than the placebo group. Similarly, Griffiths et al. (2018) reported that administering psilocybin to healthy participants alongside supported spiritual practice (which included aspects of mindfulness meditation) led to greater improvements in their mood, positive attitudes toward self and life, and prosocial attitudes and behaviors in comparison to a group who received psilocybin without the additional supported spiritual practice at 6-months follow-up. Accordingly, adding mindfulness elements into clinical trials of psychedelic therapy may be able to deliver better outcomes for patients.

Interventions combining different STEs therefore show initial yet promising results in an attempt to deliver beneficial effects on ecological wellbeing. Different varieties of STEs do not have to be pursued in an either/or fashion but appear to be able to operate in a largely complementary, potentially reinforcing manner. Future work should continue to pursue how to best combine different varieties of STEs—including those that have not been examined in detail in this paper – in order to reap the largest beneficial effects for ecological wellbeing.

### Longevity of effects

Another important area to consider when designing interventions to encourage STEs is how long the desired effects on ecological wellbeing delivered by the specific variety of STE last. Whilst STEs are states that may only last several minutes or hours, some evidence hints that they may be able to produce long lasting effects on ecological wellbeing (Miller, 2004). For example, when discussing psychedelic-induced STEs, many studies reported improvements in wellbeing and nature connectedness that lasted months to even years after the psychedelic experience itself (Griffiths et al., 2008; Schmid and Liechti, 2018; Kettner et al., 2019). With meditation too, there are some promising findings showing that the state-level neurological effects of mindful STEs can result in longer-term, trait changes in neurological functioning (Cahn and Polich, 2006; Millière et al., 2018). Experienced meditators have been shown to display reduced resting state DMN activity (Brewer et al., 2011; Hasenkamp and Barsalou, 2012; Bauer et al., 2019) along with increased resting state functional connectivity between the DMN and other brain areas such as the auditory/salience network (Kilpatrick et al., 2011). These findings suggest that the practice of mindfulness and associated meditation practices can prompt trait-level changes in brain functioning which we could speculate to represent a new way of viewing/interacting with the world over the long term with potentially positive implications for pro-environmental and pro-social behaviors. The evidence surrounding the longevity of the effects of awe and flow on wellbeing and sustainability-related variables is less developed, and we would suggest that this is a fruitful area for future work. Such research will be able to inform the time course and frequency of STEs required to achieve lasting improvements in ecological wellbeing.

### Social acceptability of the different approaches

The four varieties of STE outlined in this paper will undoubtedly have differing public perceptions. The social acceptability of each variety of STE will be important to bear in mind when deciding whether interventions should prioritize one variety over another. For instance, the wider use of psychedelics is still mostly prohibited and often has a negative reputation attached to it. While criticism can range across different areas, the potential risks of psychedelics are often the main concern for critics. According to Johnson et al. (2019), commonly cited risks include (a) anxious, dysphoric, confusing, and, less commonly, delusional acute reactions, commonly referred to as “bad trip,” (b) aggravation of symptoms in patients with psychotic disorders, and (c) temporary physiological effects in the form of modest increases in blood pressure and accelerated heart rate during the more intense stages of the STE. For these reasons, patients with a personal or family history of mental illness as well as those with significant health issues are currently excluded from psychedelic therapy (Nutt and Carhart-Harris, 2021). It is important to be aware of these potential risks when making decisions surrounding the suitability of psychedelic-induced STEs to support ecological wellbeing in future studies (Raison et al., 2022). The other three varieties of STE that we have discussed are legal, carry fewer risks and are thus more socially acceptable. If they can deliver the same beneficial effects on ecological wellbeing as psychedelic-induced STEs, they therefore currently represent more socially acceptable routes toward ecological wellbeing.

### Achieving self-transcendent experiences alongside capitalism? The importance of set and setting

Whereas the reviewed evidence suggests that STEs could hold far-reaching potential to deliver ecological wellbeing, it is worth considering the impacts of promoting STEs within societies where consumer capitalism is the dominant economic framework. Here, there are potential risks that the different types of STEs might be employed to support commercial aims, rather than those of ecological wellbeing.

For instance, mindfulness is now being marketed as a way of increasing worker productivity or reducing absenteeism (sometimes called “McMindfulness”). These uses of mindfulness to increase profits is oxymoronic when compared to its ethical, Buddhist origins, which emphasize the use of mindfulness to foster compassion and understand the causes of collective suffering (Purser and Loy, 2013). Similar problems arise for flow which, within the field of consumer psychology, is being promoted in retail environments as a way of encouraging positive brand attitudes and purchase behaviors (Hsu et al., 2013; Gao and Bai, 2014; Aboubaker Ettis, 2017). Further, the use of classic psychedelics is deeply rooted in ancient practices of indigenous populations in ceremonial contexts in countries such as Peru and Brazil, among others. Recently, however, an interest in microdosing has found its way into the creative industries and management, amongst others, with a promise to trigger creativity, deep-insights and productivity (Lant, 2017) to foster one’s career^5^ (Elf et al., 2022).

Accordingly, it will be important to consider how a capitalist market economy might try to commercialize STEs along with the positive and negative consequences this can have for ecological wellbeing. Will mindfulness still lead to improvements in wellbeing and sustainable behaviors if it is approached with the goal of increasing productivity or to sell advertising, for example? This will have important implications for how interventions are framed and promoted.

## Final remarks

We started our exploration by outlining how the features of many modern societies such as materialism, overconsumption, and a disconnect between humans and the natural world are continuously pushing our natural life-support system beyond its limits (Steffen et al., 2015b) and having detrimental effects on our psychological wellbeing. These ever growing pressures on the environment and accelerating societal problems (Eyring et al., 2021) mean that it is urgent that we find ways of transforming our mindsets and changing our behaviors that are aligned with environmental and social limits if we are to achieve ecological wellbeing.

We therefore aimed to explore the potential of novel approaches such as STEs to support that transformation. During STEs, an individual experiences a reduced salience of the individual self, alongside enhanced feelings of connection to other non-self-groups or objects. Our review of the academic literature surrounding four types of STEs suggests that STEs supported through practicing mindfulness and meditation, experiencing moments of awe and flow, and/or the careful administration of psychedelics in legal settings might hold the potential to unlock a deeper understanding of human beings that goes beyond our material existence and connects us with others and nature. For each variety of STE that we have explored, there is empirical evidence connecting the experience to both higher individual wellbeing and engagement in more sustainable attitudes and behaviors.

Nevertheless, there are still many questions that need to be answered concerning the nature of the relationship between STEs and ecological wellbeing. In particular, we need to better understand how and why they demonstrate their positive associations with both wellbeing and sustainability-related variables. In Section “Why do self-transcendent experiences support ecological wellbeing? Hypotheses to be tested,” we proposed four types of hypotheses that could be tested here covering factors including changes in self-construal, neurological determinants, increases in empathy, and increases in the personality trait of openness. In addition, there are a number of factors that need to be considered when designing STE-oriented interventions, more practically, such as their frequency, social acceptability, focus on one or multiple varieties of STE and the impact of the broader societal climate. Indeed, several of the lower-risk STEs are already being practically employed to improve factors related to ecological wellbeing. For example, mindfulness programs have been shown to successfully improve psychological wellbeing (Tang et al., 2019) whilst an awe walks intervention led to an increased sense of social connectedness and compassion (Sturm et al., 2020). Future work can explore and develop further STE-focused interventions that could facilitate change at scale, including those which focus on forms of STEs not covered in this paper such as gratitude (Chen et al., 2022).

Erich Fromm believed that humans hold the capacity to have unanticipated experiences that can bring profound insights and genuine happiness. He held that “[w]hether it be the fresh and spontaneous perception of a landscape or the dawning of some truth as a result of our thinking, or a sensuous pleasure that is not stereotyped, or the welling up of love for another person” (Fromm, 1942: 224f.). He was sure that “in these moments we all know what a human life could be if the experiences were not such rare and uncultivated occurrences” (ibid). We believe that, based on the evidence outlined in this paper, STEs have the potential to act as a catalyst for new ways of being in the world that support ecological wellbeing.

## Author contributions

AI and PE conceived the ideas presented in the manuscript and drafted the manuscript. AI, PE, and TJ revised the manuscript. All authors contributed to the article and approved the submitted version.

## Footnotes

1 The World Health Organization reports that 264 million people on the planet are depressed; depression is the third most costly disability worldwide https://www.who.int/news-room/fact-sheets/detail/depression.

2 In addition to these two core components, several additional phenomenological features are common during STEs. These include a sense of sacredness of the experience and a sense that the STE provides an encounter of ultimate reality and therefore deep meaning, sometimes referred to as noetic quality (see also Yaden et al., 2017). STEs are also often marked by a sense of positivity, tranquility, and tenderness, as well as a sense of ineffability (Stace, 1960).

3 Functional connectivity (FC) refers to the extent to which two spatially distinct brain regions show a statistical relationship in their activities.

4 Whilst a reduction in functional connectivity within the DMN has been reported by many studies across the different STEs, there are a small number of studies that have reported increased functional connectivity within certain subcomponents of the DMN in experienced meditation practitioners and those with higher levels of trait mindfulness during restful states (Shaurya Prakash et al., 2013; Taylor et al., 2013). These observed increases in functional connectivity within the DMN (during restful states rather than the STE itself) are suggested to reflect enhanced ability to intentionally ‘think-nothing’ (Jao et al., 2016).

5 Please note that legal studies exploring the effects and potential of microdosing (e.g., Szigeti et al., 2021) are still limited and most microdosing remains illegal.
